# Frizzled-7 expression during early cardiogenesis of *Xenopus laevis* embryo

**DOI:** 10.1186/1471-2164-15-S2-P17

**Published:** 2014-04-02

**Authors:** Muhammad Abu-Elmagd, Grant Wheeler

**Affiliations:** 1 Centre of Excellence in Genomic Medicine Research, King Abdulaziz University, 80216 Jeddah 21589, Kingdom of Saudi Arabia; 2 Minia University, Faculty of Science, Zoology Department, Minia, Egypt; 3 University of East Anglia, School of Biological Sciences, Norwich, NR4 7TJ, UK

## Background

The vertebrate heart makes its first sign of appearance at the gastrulation. Gene regulatory network controlling heart development is still not completely understood. Wnt signaling plays a pivotal role in orchestrating different aspects of early and late embryonic development including heart formation. Wnt ligands through canonical and non-canonical pathways have been shown to be essential for cardiac specification and morphogenesis. For example, Wnt3A and Wnt8 found to be capable of inhibiting endogenous heart induction, antagonizing Dkk-1 and Crescent and sufficient to induce heart formation in non-cardiogenic ventral marginal zone mesoderm [1]. Wnt receptors, Frizzleds, are seven-transmembrane proteins to which Wnts bind to their extracellular part, the cysteine-rich domain (CRD) [2]. Little is still known about the mechanism of Wnt-Frizzled receptors signaling during cardiogenesis. The Wnt receptor, Frizzled-7 (fzd7), has been shown by us and others to play important roles in different embryonic developmental contexts including neural crest induction [3] and convergent extension movements during gastrulation [4]. Xfz7 expression pattern during cardiogenesis in comparison to other early heart markers has not been yet investigated. In the current study, we have analyzed Xfzd7 expression in relation to that of XNkx2.5, XTroponin1-C, XGata-4, -5 and -6 and show its co-localization with these heart markers. We are aiming by this analysis to provide some clues about gene expression profile during cardiogenesis through which we might be able to shed some light on the cause of abnormal cardiac development that are Wnt-related.

## Materials and methods

*Xenopus laevis* embryos were staged according to the normal time table of Nieuwkoop and Faber [5]. Riboprobes of Xfzd7, XNkx2.5, XTroponin-1C, Gata-4, -5 and -6 were synthesized. Single and double *in situ* hybridisation was carried out according to the methods described by Harland [6] and Knecht, *et al. *[7]. Embryos were frozen sectioned and imaged using Leica microscopes with Axiovision imaging software.

## Results

Our analysis showed that Xfzd7 is strongly expressed in the heart. Using double *in situ* hybridisation, Xfzd7 expression showed overlapping with that of early heart specific markers including XNkx2.5, XTroponin-1C, Gata4, Gata5 and Gata6. Xfzd7 transcripts were also detected in the lateral plates of mesoderm.

## Conclusions

Our results suggest that strong Xfzd7 expression in the heart could add some clues on gene expression profile in general during Xenopus heart formation and shows that Xfzd7 could be used as an early heart marker. This might lead to understand some causes of abnormal cardiac development in general especially those that are Wnt-related.
